# Dynamic brain-heart-gut coupling during sleep: a continuous physiological signal analysis

**DOI:** 10.3389/fnins.2025.1594759

**Published:** 2025-05-26

**Authors:** Guojing Wang, Hongyun Liu, Shijing Wu, Xiaohua Yu, Weidong Wang

**Affiliations:** ^1^Division of Medical Innovation Research, Chinese PLA General Hospital, Beijing, China; ^2^Key Laboratory of Biomedical Engineering and Translational Medicine, Ministry of Industry and Information Technology, Chinese PLA General Hospital, Beijing, China; ^3^School of Biological Science and Medical Engineering, Beihang University, Beijing, China

**Keywords:** brain-heart-gut coupling, sleep, maximal information coefficient, continuous physiological signals, bowel sounds

## Abstract

**Background:**

Investigating brain-heart-gut coupling during sleep is crucial for understanding the coordinated regulatory mechanisms of multiple systems during sleep. Non-invasive continuous physiological signal acquisition techniques have been widely applied in brain-heart dynamic assessment. However, current research on gut function primarily focuses on gut microbiota, with a lack of systematic investigation into the macroscopic dynamic changes of gut function. This study, therefore, based on multiple non-invasive physiological signals, aims to explore the dynamic changes and underlying mechanisms of brain-heart-gut coupling during sleep.

**Methods:**

The study enrolled 24 healthy subjects, and collected electroencephalogram (EEG), electrocardiogram (ECG), and bowel sounds (BS) signals during sleep. Through signal processing and spectral analysis, power spectral values of each physiological signal in different frequency bands were extracted. The maximal information coefficient (MIC) method was employed to dynamically monitor and quantitatively analyze the coupling strength of brain-heart-gut during sleep.

**Results:**

The study revealed that the strength of brain-heart-gut coupling significantly varied with sleep stages, showing a gradual weakening trend as sleep deepened. In terms of heart-gut coupling (HGC), the coupling strength between the very low frequency (VLF) band of heart rate variability (HRV) and all BS-derived power sequences was significantly lower than other HRV frequency bands. Regarding brain-heart coupling (BHC), the EEG-beta band showed distinct sleep-stage-dependent coupling characteristics with HRV frequency bands, while the EEG-delta band exhibited higher coupling strength with HRV bands during non-rapid eye movement (NREM) sleep. Additionally, the coupling strength of HGC was significantly higher than that of BGC.

**Conclusion:**

This study successfully achieved quantitative assessment of brain-heart-gut coupling during sleep based on continuous physiological signals, revealing specific patterns of coupling strength changes across different sleep stages. This research provides new methodological support for the diagnosis of sleep disorders and functional bowel diseases, holding significant theoretical value and clinical application prospects.

## 1 Introduction

Sleep is a vital component of human physiological function, playing a pivotal role in the process of physical recovery, memory consolidation, and the coordinated functioning of multiple physiological systems. Its regulatory mechanisms involve complex interactions between the central nervous system (CNS), the autonomic nervous system (ANS), and other peripheral nervous systems. In recent years, the development of network physiology has established a new paradigm for investigating dynamic couplings across multiple physiological systems (Ivanov, 2021). Additionally, the advent of multimodal non-invasive physiological signal acquisition technologies has engendered novel research methodologies for the continuous evaluation of physiological systems during sleep. Such signals, which include electroencephalogram (EEG) (Zhuravlev et al., 2023), electrocardiogram (ECG) (Wang T. et al., 2022) and respiratory signals (Bester et al., 2023), have facilitated the exploration of brain-heart coupling (BHC) (de Zambotti et al., 2018), cardiorespiratory analysis (Bester et al., 2023) and brain-to-brain coupling (Zhuravlev et al., 2023) mechanisms. Of these, BHC has attracted considerable attention due to its role in assessing the dynamic connection between the CNS and the cardiac ANS. This field has yielded more in-depth results, including that during the transition from light to deep sleep stages, BHC exhibits a significant decreasing trend (Faes et al., 2014, 2015; Abdalbari et al., 2022). Moreover, BHC during sleep is a dynamic physiological process characterized by multiscale properties and nonlinear features. In this process, stable bidirectional interactions form between beta waves and cardiac vagal nerve activity, with nonlinear dynamics—particularly delta wave-related interactions—playing a key role in deep sleep (Faes et al., 2014, 2015; Hartmann et al., 2021; Vergara et al., 2025). These studies have not only deepened our understanding of the physiological mechanisms of sleep, but also provided new ideas for the diagnosis and treatment of sleep disorders (Whitehurst et al., 2022).

However, a growing body of research (Schemann et al., 2020; Wang Z. et al., 2022) has demonstrated that the regulatory mechanisms of sleep are intricately linked not only to CNS and ANS, but also exhibit multifaceted interactions with the gastrointestinal system. And this interaction not only reveals the important role of sleep in memory formation, but also provides new perspectives for understanding sleep disorders and cognitive decline. Furthermore, the dynamics of the gut (including intestinal peristalsis and emptying function) and the enteric nervous system (ENS) are also bi-directionally regulated with the CNS and ANS through the gut-brain axis (Mayer et al., 2023). The CNS exerts its influence over intestinal function by means of the hypothalamic–pituitary–adrenal axis (HPA axis) and the vagus nerve, while the ANS plays a pivotal role in the precise regulation of intestinal motility through the sympathetic and parasympathetic nerves. Research has demonstrated a close association between disorders of gut dynamics and dysregulation of brain-gut interactions (Orr et al., 2020; Xu et al., 2022) and a variety of sleep disorders. For instance, the circadian rhythm of the gut exhibits a strong correlation with the sleep–wake cycle, and disturbances in gut motility may result in diminished sleep quality (Xu et al., 2022). The gut microbiota has been demonstrated to influence neurotransmitter synthesis through metabolites (e.g., short-chain fatty acids), which in turn regulate sleep (Schoch et al., 2022; Wang Z. et al., 2022). However, there is a paucity of research on the interaction between gut dynamics and the cardio-cerebral system during sleep.

Recent studies in the field of gastrointestinal research have predominantly focused on the analysis of gut bacteria. However, the study of gut bacteria poses significant challenges in terms of achieving noninvasive continuous assessment of gut function. Noninvasive multi-electrophysiology has been shown to be a promising method for exploring the mechanisms of gut-brain coupling in physiological and pathological situations (Jeanne et al., 2023). The available methods for noninvasive continuous assessment of bowel function primarily encompass abdominal electromyography (Orr et al., 1997) and bowel sound (BS) monitoring (Haase et al., 2015; Du et al., 2019; Jeanne et al., 2023). However, the abdominal electromyography method is susceptible to significant noise interference, which imposes certain limitations on its application in the assessment of bowel function (Jeanne et al., 2023). Conversely, the BS method has been validated by several studies for its effectiveness in assessing bowel motility (Ficek et al., 2021; Wang G. et al., 2022). Furthermore, sleep offers optimal environmental conditions for the acquisition of BSs, as the quiet state during sleep and the almost stationary posture of the subject are conducive to the acquisition of high-quality BS signals (Ficek et al., 2021; Wang et al., 2023).

With regard to the selection of physiological signal coupling methods, a variety of techniques have been extensively employed to evaluate the correlation between physiological signals in BHC studies. Conventional linear methods, including Pearson’s correlation coefficient and coherence analysis, are capable of capturing linear dependencies between signals. However, these methods are constrained in their ability to process nonlinear and non-stationary physiological signals. To address this challenge, researchers have proposed information theory-based methods, including mutual information (MI) and transfer entropy (TE). Whereas, MI exhibits inherent limitations, including sensitivity to binning strategies (Bonita et al., 2014; Liang et al., 2021) and reduced robustness under low signal-to-noise conditions, where nonlinear coupling relationships may become undetectable (Liang et al., 2020; Xi et al., 2022). TE suffers from the curse of dimensionality in practical applications, as it requires estimating joint probability distributions conditioned on historical states, leading to exponentially increasing computational demands for large networks or long memory systems (Tian et al., 2024). TE also demands prohibitively long stationary time series for accurate estimation and is prone to false coupling in partially observed systems or those with hidden variables (Liang et al., 2020). The Maximal Information Coefficient (MIC) (Reshef et al., 2011), a nonlinear statistical method based on mutual information, has been shown to be capable of capturing linear and nonlinear dependencies between signals, while being insensitive to data distribution, thus rendering it suitable for the analysis of high-dimensional, non-stationary signals. The MIC algorithm extends the traditional concept of mutual information by adopting a more flexible gridding method to measure the degree of coupling between two variables. Furthermore, the fairness property of MIC ensures that different functional relationships obtain similar measurements at the same noise level (Liang et al., 2021). In the analysis of the coupling of continuous physiological signals, MIC is most commonly used to study the coupling between cortical EEG and surface EMG (Liang et al., 2020, 2021; Tian et al., 2024), and of course, it has also been applied to the study of the interaction between EEG and ECG (Valenza et al., 2016; Catrambone et al., 2019). In the course of the MIC calculation process, in order to enhance its robustness, optimize its partitions and circumvent unwarranted mesh refinement, researchers have implemented numerous enhancements, ranging from AppMIC (Reshef et al., 2011) to ChiMIC (Chen et al., 2016) to BackMIC (Cao et al., 2021), which have bolstered the reliability and applicability of MIC in the analysis of physiological signals.

In this paper, we established a unified interaction network integrating the brain, heart, and gut systems within the network physiology framework. Specifically, we employed synchronous acquisition of EEG, ECG, and BS signals during sleep, with inter-system coupling strength quantified using the BackMIC method. To accurately elucidate macroscopic dynamic patterns of multi-system coordination, we implemented rigorous controls for potential confounders including sleep staging, body movements, and autonomic activity. This study not only revealed the physiological mechanisms underpinning multi-system synergy during sleep, but also provided novel insights and methodologies for the diagnosis of sleep disorders and functional intestinal diseases.

## 2 Materials and methods

### 2.1 Participants

A total of 24 individuals were enrolled for overnight data collection (refer to Table 1). All participants were identified as good sleepers, defined by the absence of a history of insomnia, a sleep onset latency of <15 min, an average total sleep duration of approximately 8 h, and fewer than two awakenings after sleep onset (Ramlee et al., 2017). This study employs a combined subjective and objective approach to assess sleep quality. In the experimental procedure, participants first complete a subjective self-assessment of their sleep status. Subsequently, professional technicians perform sleep stage annotation based on polysomnography (PSG) data and conduct objective quantitative analysis of sleep quality using video monitoring recordings. Participants who fail to meet the sleep quality requirements are excluded. Eligibility criteria excluded individuals with a history of cardiovascular, neurological, or gastrointestinal disorders to ensure no potential disruptions in gut function. In addition, to eliminate the interference of obstructive sleep apnea (OSA) with the study results, this study synchronously collects multiple physiological parameters such as oronasal airflow and thoracoabdominal respiratory movements through PSG equipment. Professional technicians annotate the presence of any sleep events to ensure that the final included data comply with the study’s inclusion criteria. Participants were instructed to consume minimal or no dinner and to avoid medications or foods that could interfere with sleep. The study was conducted at the Sleep Monitoring Center of the First Medical Center of the Chinese People’s Liberation Army General Hospital. Approval was obtained from the Medical Ethics Committee of the Chinese People’s Liberation Army General Hospital (approval number: S2022-341-01), and all participants provided written informed consent after being fully informed of the experimental procedures.

**Table 1:** Basic information of participants.

Demographic and sleep parameters	Mean ± SD
Male/female	12/12
Age (y)	29 ± 4
Body mass index	22.89 ± 4.08
Total sleep time (min)	464.44 ± 67.44
Fraction of awake	0.17 ± 0.14
Fraction of LS	0.48 ± 0.10
Fraction of DS	0.19 ± 0.06
Fraction of REM	0.16 ± 0.06

### 2.2 Data acquisition

PSG was performed to record EEG signals (F3/M2, F4/M1, C3/M2, C4/M1, O1/M2, O2/M1; electrode impedance ≤5kΩ) according to the international 10–20 system, along with synchronized ECG (right arm/left arm leads) (Chase et al., 2022; Wang T. et al., 2022). All PSG data were recorded using the Embla^®^ system (Natus Medical Incorporated, Pleasanton, USA), with a uniform sampling rate of 500 Hz for both EEG and ECG signals. Participants also wore thoracic and abdominal respiratory belts along with a nasal cannula for respiratory monitoring. Additionally, other PSG-required signals were recorded, including leg electromyography, oxygen saturation, and body position/movement information. The start and end times of sleep monitoring are recorded synchronously with the laboratory light switch times (with sleep onset marked as “lights off” and sleep termination as “lights on”), serving as anchor points for sleep stage analysis.

Sleep stages were determined by trained experts based on 30-s epochs in accordance with the standard guidelines of the American Academy of Sleep Medicine (AASM) (Berry et al., 2020), distinguishing the awake state (stage Awake), light sleep (LS) with stage N1 and N2, deep sleep (DS) with stage N3, and rapid eye movement (REM) sleep (Ma et al., 2024). Here, the LS and DS stages are collectively referred to as non-rapid eye movement (NREM) sleep.

Sleep EEG and ECG signals were collected using the PSG device, while sleep BSs were recorded using a self-developed BS recorder (Wang et al., 2023). The BS recorder utilized a Knowles SiSonic MEMS microphone (SPU1410LR5H-QB, with bottom holes) for sound acquisition. The device featured a dual-channel sound acquisition system operating at a sampling rate of 8,000 Hz. The BS channel captured raw bowel sounds through a front-facing microphone chip on the circuit board, while the noise (NS) channel recorded external environmental noise via a rear-facing microphone chip. To minimize interference from heart sounds, the BS recorder was strategically placed on the lower right abdominal surface to obtain BS signals.

### 2.3 Data processing

**Figure 1:**
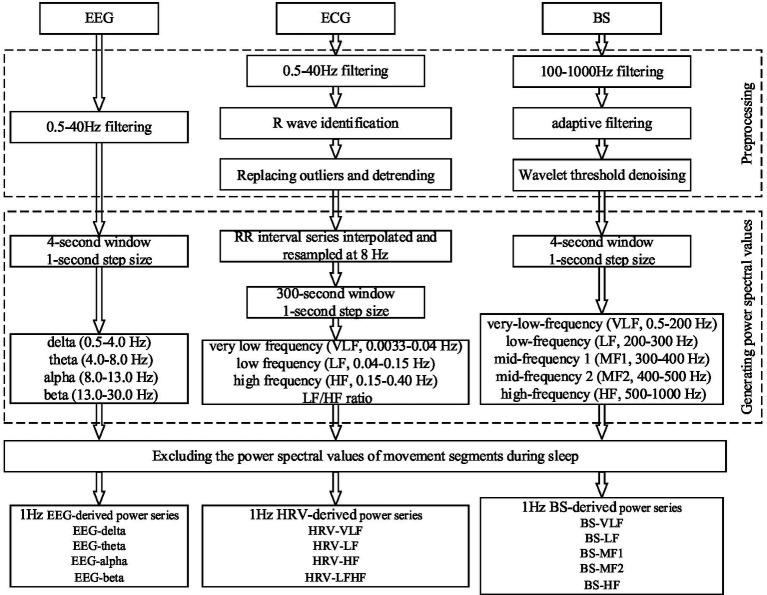
Data processing block diagram.

The objective of data processing is to enhance the signal quality and extract the frequency power series of sleep EEG, ECG and BS signals. Prior to the implementation of data processing, it is imperative to acknowledge that BSs become predominantly obscured during physical movements. Furthermore, sleep EEG signals are vulnerable to interference from limb movements (Lyu et al., 2023). Additionally, we excluded data segments containing body-turning movements and subsequently performed stratified MIC calculations based on sleep stages which can better control for motion artifacts and sleep-stage-specific effects. To mitigate the impact of body movements on signal integrity, the Activity channel of the PSG system was utilized to detect body movements through accelerometry and the threshold was manually adjusted to 0.2, enabling the precise identification of movement segments and the subsequent exclusion of data from these segments (Kuo et al., 2016). Figure 1 shows the data processing diagram.

#### 2.3.1 Sleep EEG

Six-channel EEG signals referenced to the bilateral mastoids (M1, M2) were collected with the PSG device. Additionally, studies have demonstrated that signals associated with CNS and cardiorespiratory coupling exhibit significant occipital tendance, thereby suggesting that the occipital region may serve as a pivotal brain area for receiving and processing the interactions of CNS-cardiorespiratory network system information (Yang et al., 2021). Consequently, this study had opted to utilize the occipital O2 channel EEG signal as the primary basis for assessing brain electrical activity. The processing of EEG signals was undertaken using a fourth-order Butterworth bandpass filter with a frequency range of 0.5–40 Hz, an approach that had been demonstrated to be effective in the removal of low-frequency drift and high-frequency interference (Hartmann et al., 2021; Wang T. et al., 2022; Lyu et al., 2023). Subsequently, power spectral density was computed using a 4-s sliding window with 1-s overlap, generating 1-Hz resolution power time series (Valenza et al., 2016). Specifically, the Welch periodogram method was utilized to calculate the absolute power spectral values (μV^2^) for the following frequency bands: delta (0.5–4.0 Hz), theta (4.0–8.0 Hz), alpha (8.0–13.0 Hz), and beta (13.0–30.0 Hz), respectively. Consequently, the EEG power series of EEG-delta, EEG-theta, EEG-alpha and EEG-beta could be obtained. During sleep, the activity of different frequency bands of EEG reflects distinct physiological functions. The delta band, a hallmark of slow-wave sleep, becomes more prominent during deep sleep stages and is closely associated with the body’s physiological restoration processes. Theta band activity is notably enhanced during REM sleep and light sleep stages, with this rhythm believed to be linked to neural activity in the limbic system, particularly the hippocampus. The alpha band is most pronounced during wakefulness with eyes closed, while during sleep, it accompanies the characteristic sleep spindles of N2 stage. The beta band typically reflects alertness and cognitive processing, and its increased activity during REM sleep may suggest active neural information processing related to dreaming (Mann et al., 1993).

#### 2.3.2 Sleep ECG

Since the study of the regulatory function of the ANS during sleep requires the RR interval (RRI) which is based on ECG to realize heart rate variability (HRV) analysis (Cheng et al., 2023). Therefore, the processing of sleep ECG should first identify RRI and then calculate the power spectrum. The raw ECG signal was initially filtered with a fourth-order Butterworth bandpass filter (0.5–40 Hz) to eliminate noise interference (Wang T. et al., 2022). The QRS complex detection was achieved by implementing a Haar wavelet transform in conjunction with an adaptive thresholding method. This approach identified local maxima within the QRS region and the point of maximum amplitude was confirmed as the R-wave peak. Following the R-wave identification, a 21-point moving window was applied to calculate the mean and standard deviation centered on each data point. Abnormalities were detected by comparing the standardized deviation with a predefined threshold (set at 4) (Pilia et al., 2021). Data points exceeding this threshold were replaced with the corresponding mean value, while those below the threshold were retained (Lyu et al., 2023). Subsequently, the RRI was detrended using the db12 wavelet transform. The detrended RRI series was then uniformly interpolated and resampled at 8 Hz. For power spectral density estimation, the autoregressive model with the Burg algorithm was employed, using a 300-s sliding window with a 1-s overlap. To mitigate potential confounding effects from ANS activity, we performed HRV frequency-band stratification analysis (Porta et al., 2017). Specifically, relative power values were computed for the following frequency bands: very low frequency (VLF, 0.0033–0.04 Hz), low frequency (LF, 0.04–0.15 Hz) and high frequency (HF, 0.15–0.40 Hz). Additionally, the LF/HF ratio was calculated. These computations yielded the 1 Hz HRV power series—HRV-VLF, HRV-LF, HRV-HF and HRV-LFHF. During sleep, the VLF component may reflect the fundamental rhythmic characteristics of sleep-disordered breathing and periodic limb movements. The power spectra of the LF band and HF band are closely associated with the regulatory functions of the sympathetic nervous system and parasympathetic nervous system, respectively. Analysis of the LF/HF ratio can be used to assess dynamic changes in autonomic nervous function across different sleep stages. Notably, during the transition from NREM sleep to REM sleep, an increase in this ratio often indicates enhanced sympathetic activity, demonstrating the sensitivity of this metric in capturing subtle variations in cardiac autonomic regulation throughout sleep stages (Malik, 1996; Lyu et al., 2023).

#### 2.3.3 Sleep BSs

The processing of sleep BSs was analogous to that of sleep EEG in that noise processing was executed prior to the acquisition of the power series. The noise processing of sleep BSs principally comprised band-pass filtering, adaptive filtering, and wavelet threshold denoising (Wang et al., 2023). Initially, band-pass filtering of 100–1,000 Hz was employed to eliminate out-of-band noise in the BS signal (Wang G. et al., 2022; Wang et al., 2023). Then adaptive filtering, based on the dual-channel configuration of the device, was used to remove environmental noise. This study employed the normalized least mean square (NLMS) algorithm to achieve adaptive noise removal, setting the filter length to 64 and the step size to 0.001 (Al-Naggar and Al-Udyni, 2018; Wang et al., 2023). Finally, wavelet threshold denoising was used to suppress noise components concentrated in the detailed components of the wavelet decomposition. The wavelet basis was selected as sym6, the number of decomposition layers was determined to be 6, and the threshold was computed using the Birge-Massart algorithm to achieve efficient threshold denoising (Sidhik, 2015; Wang et al., 2023). For the denoising-optimized sleep BSs, the relative power spectral densities of the following frequency bands were estimated using the Welch periodogram method withing a sliding window of 4-s and the overlap of 1-s: very-low-frequency (VLF, 0.5–200 Hz), low-frequency (LF, 200–3 00 Hz), mid-frequency 1 (MF1, 300–400 Hz), mid-frequency 2 (MF2, 400–500 Hz), and high-frequency (HF, 500–1,000 Hz). The power series of different frequency bands corresponding to the sleep BSs—BS-VLF, BS-LF, BS-MF1, BS-MF2, and BS-HF—were obtained, respectively.

### 2.4 MIC

To analyze the coupling relationships between the brain, heart, and gut during sleep, we employed MIC for assessment. The MIC explores all possible grid partitions of two variables within a finite dataset to search for the maximal mutual information, which is then normalized. Specifically, for a pair of variables (X,Y), where X∈Rn, Y∈Rn, the MIC of X and Y is defined as Equation 1 (Cao et al., 2021):


(1) $$\text{MIC}(X,Y)=\max_{n_{x}\times n_{y}\leq B(n,\alpha )}\{\frac{\max_{G}(I_{G}(X,Y))}{\log_{2}\min(n_{x},n_{y})}\}$$

where $n_{x}$ and $n_{y}$ are the number of bins on the x-axis and y-axis, respectively. G represents a $n_{x}\times n_{y}$ grid on (X,Y), $I_{G}(X,Y)$ denotes the mutual information under the grid G, and B(n,α) is a function of data size n and is equal to $n^{\alpha }\;(0<\alpha <1)$, which limits the maximum number of bins. The normalization term $\log_{2}\min(n_{x},n_{y})$ ensures that MIC lies in the range of 0–1. The closer the value was to 1, the stronger the correlation between the two variables is.

Although traditional MIC outperforms nonlinear methods such as mutual information in association detection, its grid search strategy still affects coupling results and computational complexity. To address these problems, this study adopted an improved algorithm—BackMIC—for estimating MIC (Cao et al., 2021). The BackMIC algorithm terminates grid optimization through a *χ*^2^ test. Specifically, given an optimal segmentation point, if the *p*-value of the *χ*^2^ test for this segmentation point is below a given threshold (here set to 0.01), the segmentation point is considered valid, and the BackMIC algorithm continues searching for the next optimal segmentation point; otherwise, the algorithm stops the search process. Unlike the AppMIC and ChiMIC algorithms, this algorithm adds a reverse search process on the equidistant axis to eliminate the limitations of equidistant partitioning. Compared to the AppMIC and ChiMIC algorithms, the BackMIC algorithm demonstrates superior performance in measuring the correlation between independent and dependent variable pairs.

In the process of assessing brain-heart-gut coupling during sleep, for the EEG-derived, HRV-derived, and BS-derived power series obtained during sleep, a 5-min window (Hermann et al., 2024) was used to calculate the MIC, thereby evaluating brain-heart coupling (BHC), brain-gut coupling (BGC), and heart-gut coupling (HGC) respectively.

### 2.5 Statistical analysis

Statistical analyses were conducted to examine variations in MIC values across different sleep stages using IBM SPSS Statistics 25. Prior to conducting the statistical analyses, the normality of the data distribution was evaluated using the Kolmogorov–Smirnov test. Given that the majority of the samples did not exhibit a normal distribution, non-parametric tests were employed for subsequent analyses, with a statistical significance threshold set at *p* < 0.05. The results were expressed as median (interquartile range) to accurately represent the distribution characteristics of the data. For multiple comparisons between groups, the Kruskal–Wallis one-way ANOVA test was applied to all pairwise comparisons. Additionally, significance values were adjusted using the Bonferroni correction to account for multiple testing.

## 3 Results

This study collected sleep data from 24 participants, with the total duration and duration distribution of each sleep stage presented in Table 2. Through power spectral analysis of electroencephalogram (EEG), electrocardiogram (ECG), and body movement (BS) signals during sleep, combined with coupling analysis using the maximal information coefficient (MIC) method (calculating one MIC value per 5-min epoch), Table 2 also displays the total number and distribution of 5-min epochs across different sleep stages.

**Table 2:** Total sleep duration and distribution of 5-min epochs for MIC analysis.

Sleep stage	Total duration (min)	Duration distribution (Mean ± SD, min)	Total epochs	Epoch distribution (Mean ± SD)
Awake	1,760.88	73.37 ± 30.38	352	14 ± 6
LS	5,460.58	227.52 ± 59.29	1,092	45 ± 11
DS	2,050.57	85.44 ± 30.45	410	17 ± 6
REM	1,874.52	78.10 ± 34.08	374	15 ± 6
Total	11,146.55	464.44 ± 67.44	2,228	92 ± 13

By calculating the MIC between the EEG-derived, HRV-derived, and BS-derived power series across different frequency bands during sleep, the variations in BHC, BGC and HGC could be systematically evaluated during sleep.

### 3.1 Brain-heart-gut coupling across distinct sleep stages

**Figure 2:**
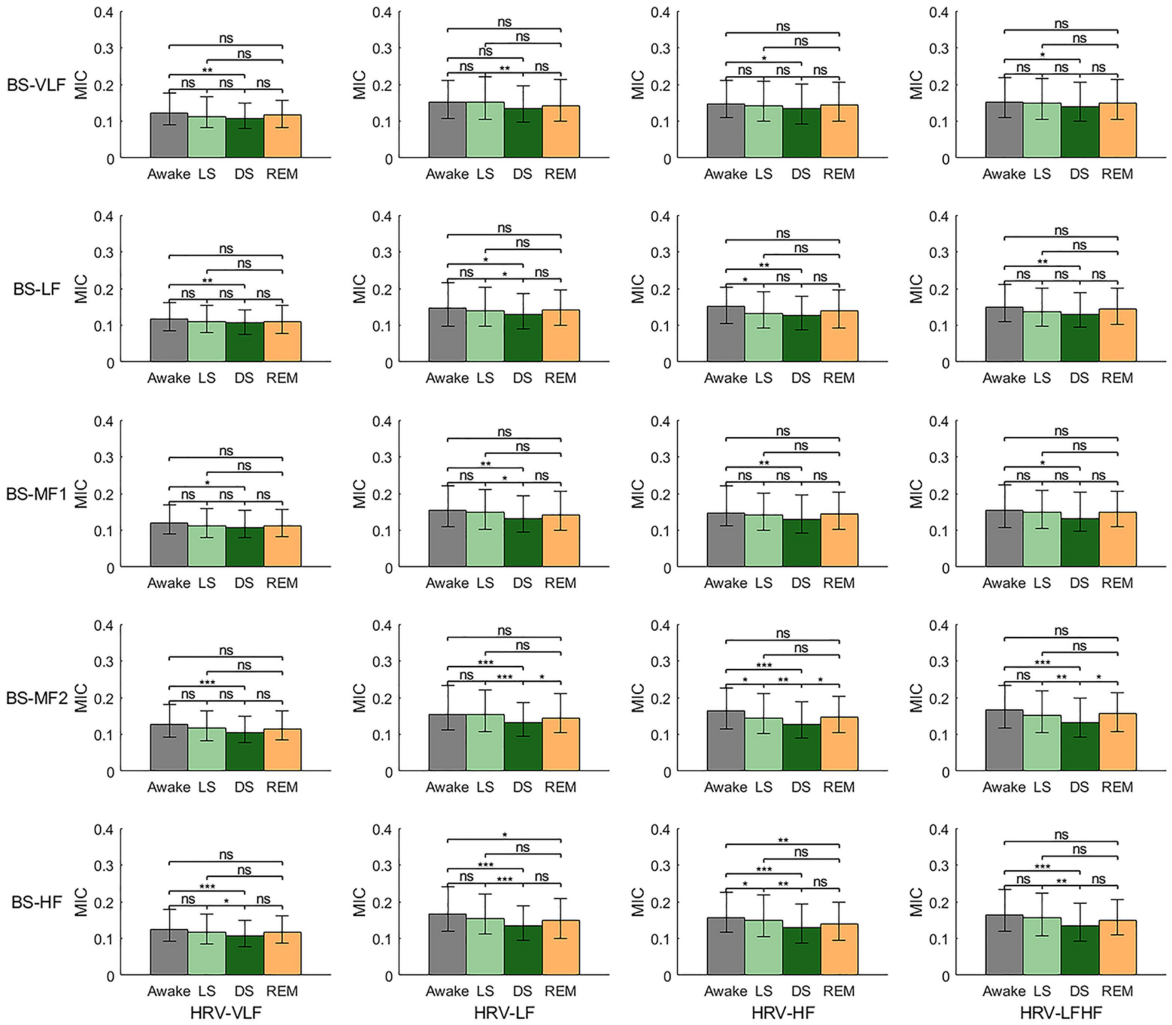
Heart-gut coupling (HGC) based on the maximal information coefficient (MIC) of HRV-derived and BS-derived power series. ns: *p* > 0.05; *p < 0.05; **p < 0.01; ***p < 0.001.

Figure 2 illustrates the MIC values between HRV-derived and BS-derived power series across different frequency bands, providing insights into HGC dynamics. Overall, HGC intensity reached its lowest value during stage DS. Detailed analysis revealed that while most frequency bands show no statistically significant differences in MIC values from stage W to LS, several exceptions existed—the MIC values between the HRV-HF and BS-LF/BS-MF2/BS-HF. From stage LS to DS, the MIC values between HRV-LF and all the BS-derived power series exhibited a declining trend. A similar trend was observed between HRV-HF and BS-HF/BS-MF2, as well as between HRV-LFHF and BS-HF/BS-MF2. Furthermore, from stage DS to REM, no statistically differences were observed in MIC values between BS-derived power series (with the exception of BS-MF2) and HRV-derived power series.

**Figure 3:**
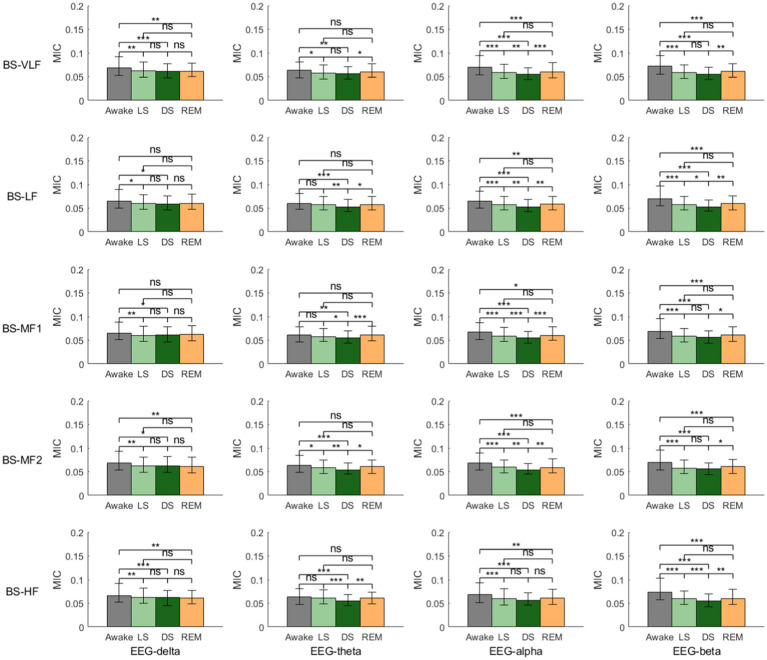
Brain-gut coupling (BGC) based on the maximal information coefficient (MIC) of EEG-derived and BS-derived power series. ns: *p* > 0.05; *p < 0.05; **p < 0.01; ***p < 0.001.

Figure 3 depicts the MIC values of EEG-derived and BS-derived power series across different frequency bands, offering an analysis of BGC. Overall, similar with HGC, BGC intensity reached its lowest value during stage DS. During the transition from stage W to LS, except EEG-theta, MIC values between all the EEG-derived and BS-derived power series showed significant decreases. From stage LS to DS, it revealed that: (1) no significant differences were observed in MIC values between EEG-delta and any BS frequency bands; (2) EEG-theta showed no statistical difference only with BS-VLF, while demonstrating decreasing trends with other BS frequency bands; (3) EEG-alpha exhibited generalized reductions in MIC values with all BS frequency bands (except BS-HF); and (4) EEG-beta maintained overall stability with BS frequency bands, but showed significant decreases with BS-LF and BS-HF. During the transition from stage DS to REM, while MIC values between EEG-delta and all BS frequency bands remained unchanged, most other frequency bands demonstrated significant rebound trends.

**Figure 4:**
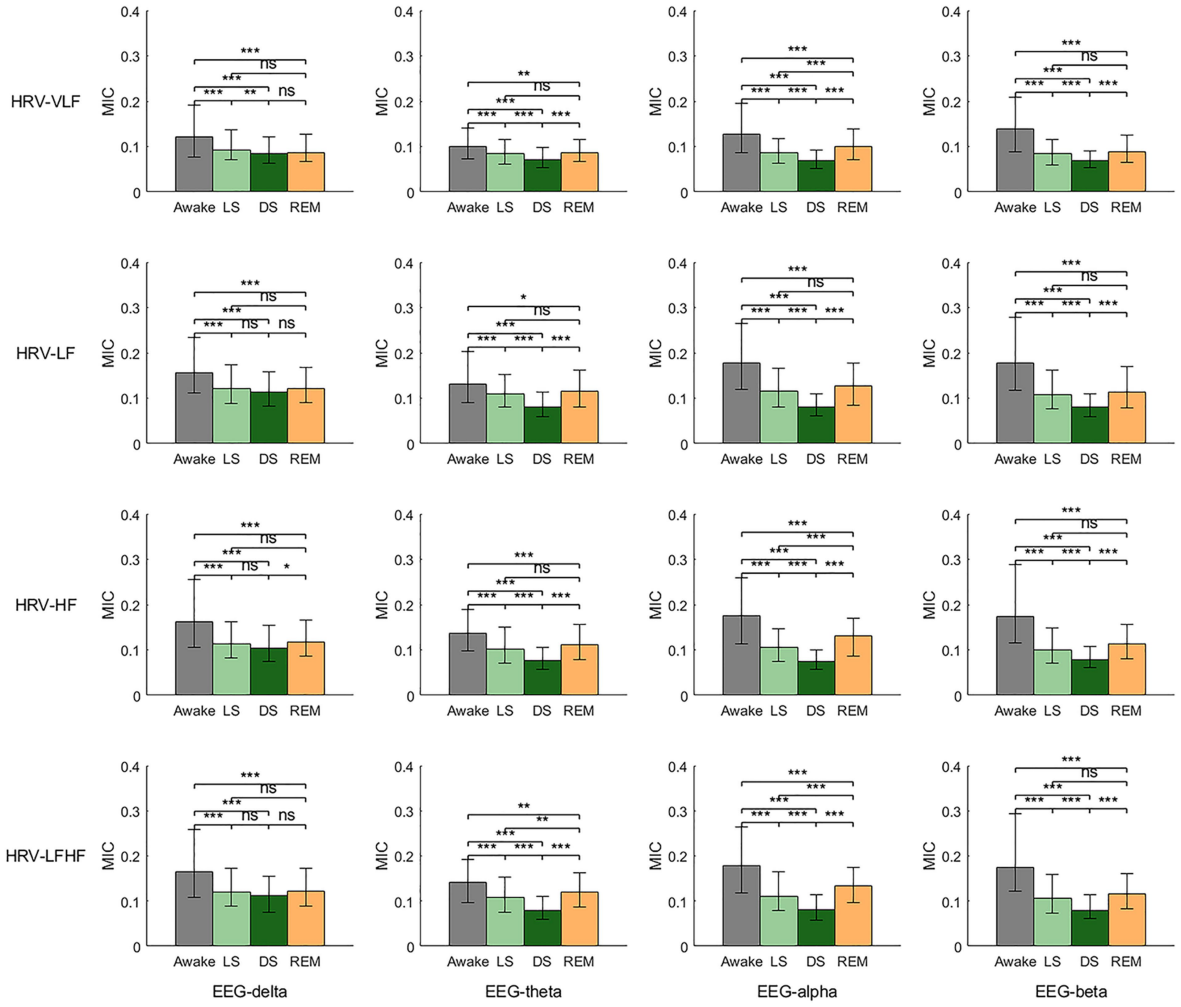
Brain-heart coupling (BHC) based on the maximal information coefficient (MIC) of EEG-derived and HRV-derived power series. ns: *p* > 0.05; *p < 0.05; **p < 0.01; ***p < 0.001.

Figure 4 presents the MIC values derived from the power series of EEG and HRV across different frequency bands, providing an analysis of BHC. Overall, the BHC group also exhibited the characteristic of MIC values reaching their lowest during stage DS. Specifically, during the transition from stage W to LS, all MIC values showed a significant decrease. In the shift from stage LS to DS, except for the MIC values between EEG-delta and HRV-LF/HRV-HF/HRV-LFHF, which showed no statistically significant differences, the remaining MIC values displayed a declining trend. During the transition from stage DS to REM, except for the MIC values between EEG-delta and HRV-VLF/HRV-LF/HRV-LF/HF, which showed no significant statistical differences, other MIC values demonstrated an upward trend. It is particularly noteworthy that the BHC group shared another consistent pattern: compared to stage W, MIC values during stage REM showed a clear decreasing trend. In addition, the coupling relationship between EEG-beta and HRV-derived power series is most consistently modulated by sleep stages, showing a continuous decrease from wakefulness to light sleep and further to deep sleep, with a rebound observed during REM sleep.

### 3.2 Brain-heart-gut coupling in each sleep stage

**Figure 5:**
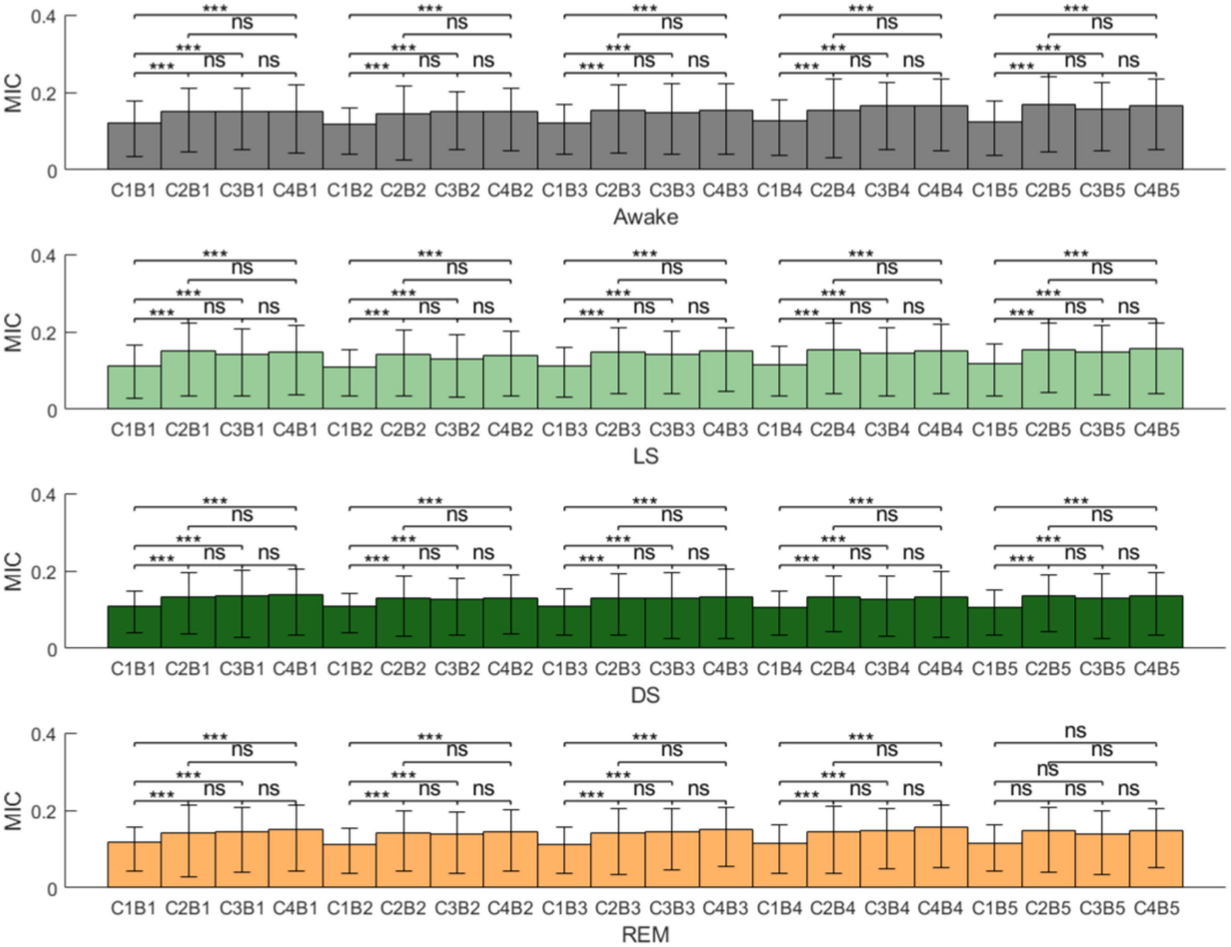
Heart-gut coupling (HGC) in different sleep stages (C1, HRV-VLF; C2, HRV-LF; C3, HRV-HF; C4, HRV-LFHF; B1, BS-VLF; B2, BS-LF; B3, BS-MF1; B4, BS-MF2; B4, BS-HF; MIC, maximal information coefficient; ns: *p* > 0.05; *p < 0.05; **p < 0.01; ***p < 0.001).

**Figure 6:**
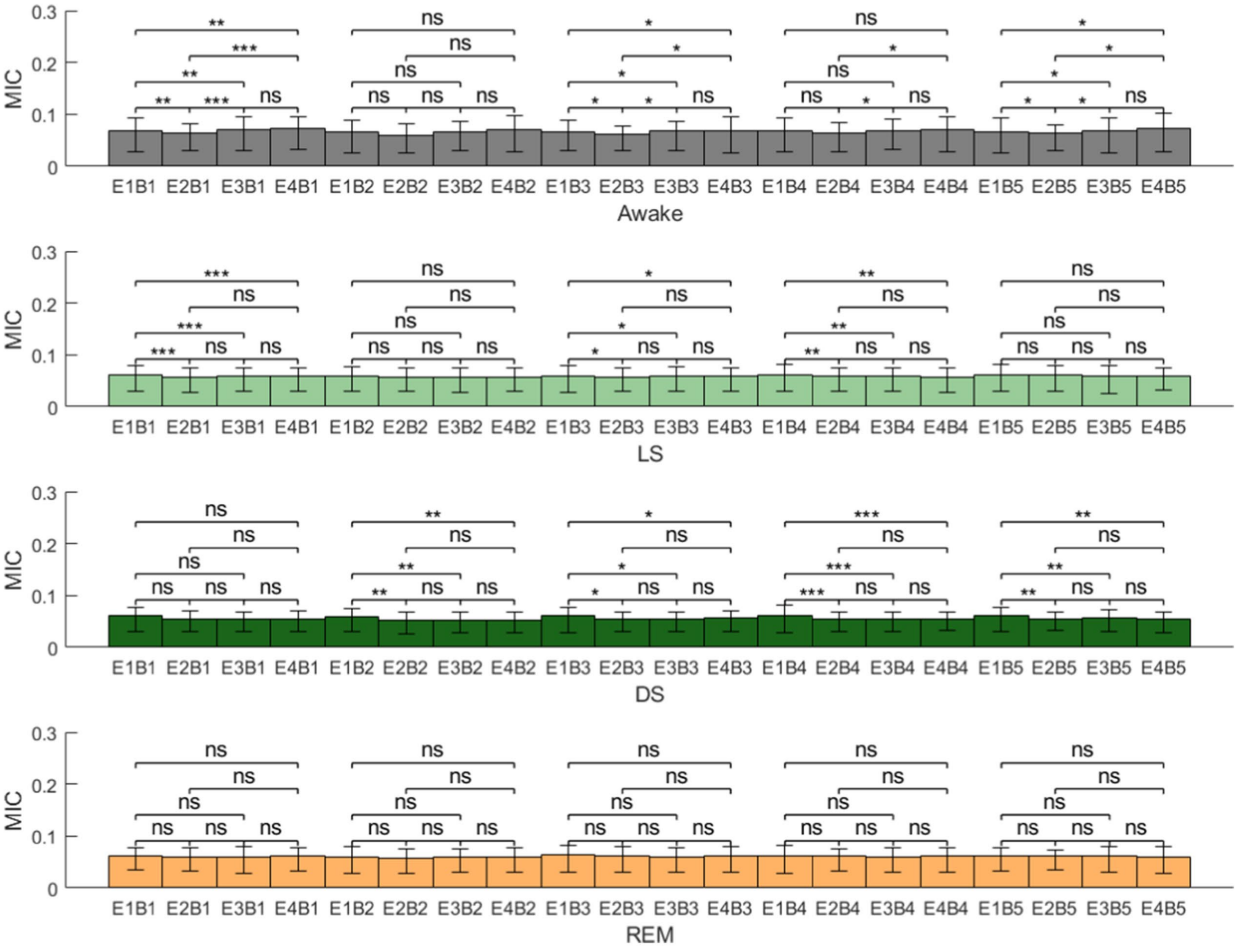
Brain-gut coupling (BGC) in different sleep stages (E1, EEG-delta; E2, EEG-theta; E3, EEG-alpha; E4, EEG-beta; B1, BS-VLF; B2, BS-LF; B3, BS-MF1; B4, BS-MF2; B5, BS-HF; MIC, maximal information coefficient; ns: *p* > 0.05; *p < 0.05; **p < 0.01; ***p < 0.001).

**Figure 7:**
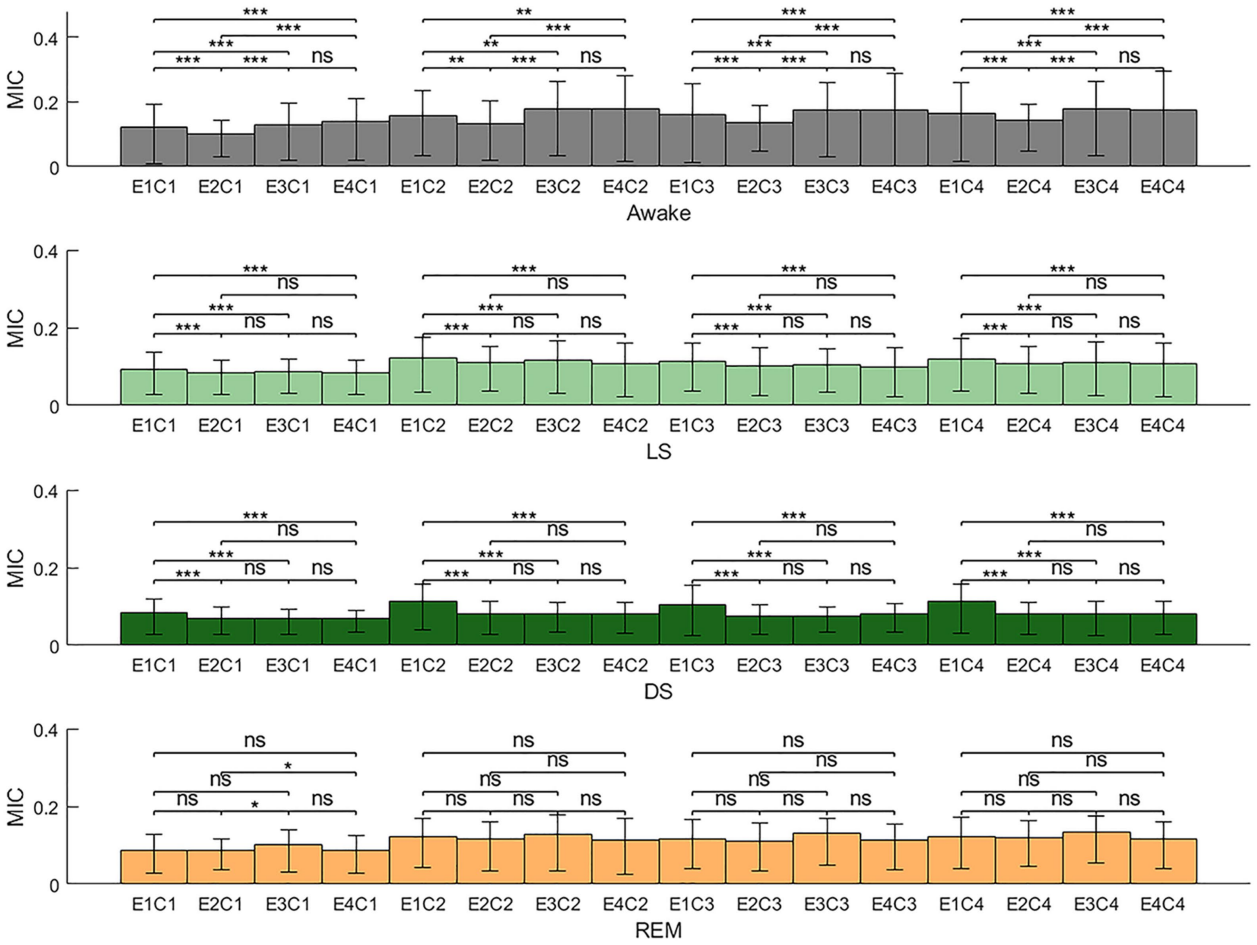
Brain-heart coupling (BHC) in different sleep stages (E1, EEG-delta; E2, EEG-theta; E3, EEG-alpha; E4, EEG-beta; C1, HRV-VLF; C2, HRV-LF; C3, HRV-HF; C4, HRV-LFHF; MIC, maximal information coefficient; ns: *p* > 0.05; *p < 0.05; **p < 0.01; ***p < 0.001).

While Figures 2–4 primarily focus on illustrating the trends of coupling strength across different sleep stages, the following Figures 5–7 provide a more detailed comparison by highlighting the coupling differences between frequency bands within each individual sleep stage.

Figure 5 consolidates the MIC values between all frequency bands of HRV and BS across different sleep stages. It was evident that across all sleep stages, the MIC between HRV-VLF and all frequency bands of the BS power series exhibit a significant decrease. In contrast, no statistically significant differences were observed in the MIC between HRV-LF/HRV-HF/HRV-LFHF and all frequency bands of the BS power series.

Figure 6 consolidates the MIC values between all frequency bands of EEG and BS across different sleep stages. Overall, BGC did not exhibit a unified pattern of variation across different sleep stages. Detailed analysis revealed that during stage LS, the coupling strength between EEG-delta and BS-derived power series was significantly higher than other EEG frequency bands, except for BS-LF and BS-HF. In stage DS, EEG-delta also demonstrated stronger coupling with BS bands compared to other EEG bands, with the exception of BS-VLF. During stage REM, however, no statistically significant differences were observed between any EEG-derived and BS-derived power series.

Figure 7 consolidates the MIC values between all frequency bands of EEG and HRV across different sleep stages, providing a comprehensive visualization of BHC dynamics in each sleep stage. The results demonstrated that during stage W, the MIC values between EEG-theta and all HRV frequency bands were relatively low, whereas the MIC values between EEG-alpha/EEG-beta and all HRV frequency bands were comparatively higher. During stage LS and DS, the MIC values between EEG-delta and HRV-derived power series were generally higher than other MIC values. No prominent patterns or statistically significant variations were observed during the REM sleep stage.

### 3.3 BGC and HGC in different sleep stages

**Figure 8:**
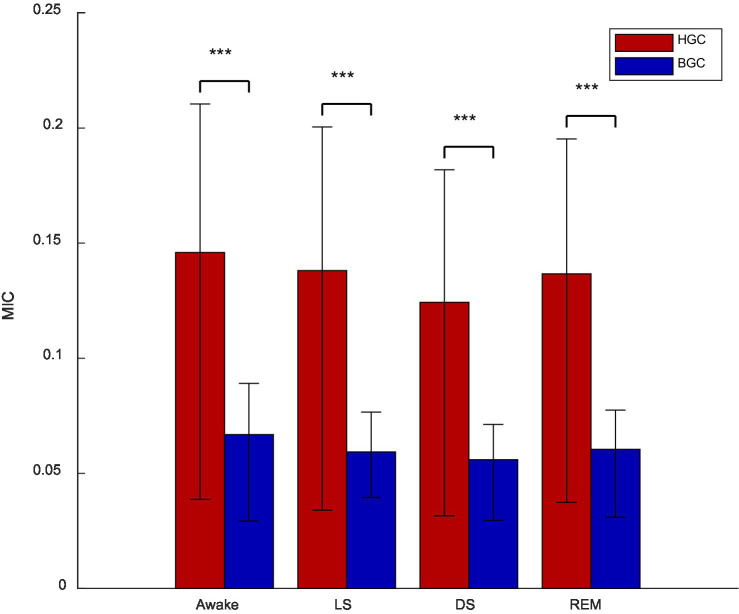
Brain-gut coupling (BGC) and heart-gut coupling (HGC) in different stages (***p < 0.001; MIC, maximal information coefficient).

Figure 8 compared all MIC values reflecting BGC with those reflecting HGC, providing an analysis of the coupling strength between the ANS (heart) and the CNS (brain) with gut motility throughout the sleep process. It was apparent that across all sleep stages, HGC was consistently greater than BGC.

## 4 Discussion

This study evaluated the brain-heart-gut coupling across different sleep stages based on simultaneously acquired sleep EEG, ECG and BS signals. The results demonstrated that: (1) brain-heart-gut coupling exhibited significant variations across sleep stages, with an overall trend of gradual weakening as sleep deepened; (2) for HGC: the HRV-VLF exhibited lower coupling with all the BS-derived power series compared with other HRV bands, and among HRV-LF, HRV-HF and HRV-LFHF showed no significant different coupling with BS-derived power series; (3) for BHC: The EEG-beta and HRV-derived power spectrum coupling exhibited the most consistent sleep-stage-dependent modulation, while EEG-delta demonstrated stronger coupling with HRV during NREM sleep; (4) HGC was generally stronger than BGC. This study provided a quantitative assessment of brain-heart-gut coupling during sleep through continuous physiological signals. This study not only realized the dynamic brain-heart-gut coupling but also offers novel insights for diagnosing and evaluating sleep disorders and functional bowel diseases.

Existing studies of brain-gut interactions have focused on the microbiota-gut-brain axis, emphasizing the exploration of interaction mechanisms at the microscopic level. In contrast, studies of brain-gut coupling based on noninvasive continuous physiological signals allow for dynamic assessment at the macroscopic level. Although some studies have attempted to assess brain-gut interactions using noninvasive methods (Huang et al., 2010; Jeanne et al., 2023), most of them have used intestinal electromyography signals to assess gut motility, which is susceptible to interference. In contrast, by obtaining more stable and reliable BS signals in the sleep environment, the present study not only realizes the dynamic macroscopic assessment of brain-heart-gut coupling, but also extends the coupling to the sleep domain, which has broader and deeper clinical significance.

The results of the study revealed significant differences in brain-heart-gut coupling across sleep stages, with the lowest coupling degree observed during deep sleep, which is consistent with previous research findings (Bashan et al., 2012; Ma et al., 2018; Rizzo et al., 2022). During deep sleep stages, the hierarchical distribution of related network connection strength reached its lowest level, potentially related to the overall relaxation of physiological systems and autonomic regulation mechanisms during sleep (Bashan et al., 2012), which was further supported by the brain-heart-gut coupling patterns observed in this study. These findings deepen our understanding of multi-system coupling during sleep.

Regarding HGC, our study indicated that the coupling strength between HRV-VLF and all the BS frequency bands is significantly lower than that of other HRV frequency bands. This suggests that the VLF component of HRV exerts relatively limited regulatory effects on intestinal motility. This finding is consistent with previous research, which indicates that the VLF band is primarily influenced by thermoregulation, hormonal regulation, and vasomotor activity rather than direct ANS regulation (Busek et al., 2005; Ali and Chen, 2023). Mechanistically, the origin of the VLF rhythm may be traced back to the activation of cardiac afferent sensory neurons, which subsequently function through feedback-feedforward loops at various levels of the cardiac intrinsic nervous system and neural circuits between the heart, extrinsic cardiac ganglia, and spinal cord (Shaffer and Ginsberg, 2017). These loops have relatively weak regulatory effects on intestinal function. In contrast, the coupling between HRV-LF/HRV-HF/HRV-LFHF and all the BS power series was significantly higher than HRV-VLF. This result meets the previous research, which has shown that HF of HRV mainly reflects cardiac parasympathetic (especially vagal) activity, LF power is influenced by both sympathetic and parasympathetic systems, and the LF/HF ratio is often used as an indicator of autonomic balance (Ali et al., 2021; Ali and Chen, 2023). Furthermore, enhanced parasympathetic activity has been shown to promote gastrointestinal motility, while increased sympathetic activity exerts inhibitory effects (Ali and Chen, 2023).

For BHC, results indicated that apart from the overall lowest coupling observed during deep sleep, the coupling relationship between the EEG-beta band and HRV-derived power series was most significantly modulated by sleep stages (Figure 4). Specifically, it showed a progressive decline from wakefulness to LS and further to DS, with a characteristic rebound during REM sleep, which aligns with existing research (Faes et al., 2014; Vergara et al., 2025). Additionally, during non-REM sleep, the EEG-delta band exhibits more prominent coupling strength with various HRV frequency bands compared to other EEG bands (Figure 7). This phenomenon underscores the pivotal role of the delta band in the nonlinear characteristics of BHC during sleep (Faes et al., 2015; de Zambotti et al., 2018; Vergara et al., 2025). However, similar to HGC, no statistically significant difference was found in the coupling strength between HRV-LF/HRV-HF and EEG in BHC. This suggests that current analytical methods cannot distinguish the differential modulation of sympathetic and parasympathetic nervous system activity on EEG, pointing to an important direction for future research. Some existing studies have indicated that changes in HRV-HF power may slightly precede EEG-delta wave fluctuations, implying that autonomic nervous system regulation might occur earlier than central nervous system transitions between sleep stages (de Zambotti et al., 2018). Future research could incorporate time-lagged information into coupling analyses, potentially yielding more definitive results.

In terms of BGC, results revealed that, similar to BHC, EEG-beta exhibited the most pronounced sleep-stage-dependent modulation in its coupling strength with various BS frequency bands compared to other EEG bands (Figure 3). Specifically, the EEG-beta/BS coupling demonstrated a characteristic “decline-rebound” dynamic pattern: decreasing from Awake through NREM sleep, followed by a rebound during REM sleep. This pattern suggests that EEG-beta also plays a state-specific role in BGC regulation mechanisms. Furthermore, Figure 6 results highlighted the predominant role of EEG-delta oscillations in BGC: EEG-delta showed significantly stronger coupling with both BS-MF1 and BS-MF2 during NREM sleep compared to other EEG bands. This finding provides additional evidence supporting the crucial role of EEG-delta oscillations in mediating nonlinear characteristics of brain–body coupling.

In addition, this study revealed a significant finding: compared to BGC, HGC demonstrated much higher coupling strength. This discrepancy may be attributed to the hierarchical characteristics of neural regulation mechanisms. The ANS has direct and immediate advantages in regulating intestinal function. Specifically, the interaction between the CNS and ENS requires mediation through sympathetic/parasympathetic pathways (Tait and Sayuk, 2021; Ali and Chen, 2023). As the core system regulating visceral organs, the ANS can rapidly act on intestinal smooth muscles and secretory cells through neurotransmitters, triggering rapid changes in intestinal physiological states (Cryan et al., 2019). This efficient neural pathway may be the key mechanism underlying stronger HGC. From an anatomical perspective, the ENS is an integral component of the ANS, sharing neurotransmitter systems (e.g., cholinergic and adrenergic systems) and rhythm regulation mechanisms with the autonomic branches responsible for cardiac function regulation (characterized by HRV). This structural similarity facilitates the formation of synchronized rhythms in the heart-gut systems, resulting in enhanced physiological coupling characteristics. In contrast, the BGC that is based on EEG involves more complex cross-hierarchical regulation. In this case, the CNS requires multi-level relays through the hypothalamic–pituitary axis and dorsal vagal complex to achieve descending regulation of the ENS. This multi-synaptic transmission pathway may reduce signal transmission efficiency, resulting in relatively weaker coupling strength.

The present study has several limitations that should be considered when interpreting the results. Firstly, the EEG analysis was limited to the occipital region, which restricted the comprehensive brain assessment. It is therefore recommended that future studies incorporate multi-channel EEG signals for more extensive evaluation of other brain regions and provide richer network node information. Secondly, the more lower frequency components of BSs were filtered out. To obtain BS signals less affected by heart sounds, data were collected near the lower right abdominal region, and a filter was used to remove signals below 100 Hz. While this effectively eliminated heart sound interference, it also removed potentially valuable information below 100 Hz, limiting observation of BS slow waves and their coupling with CNS and ANS. Thirdly, while the current study mitigated certain confounding effects through spectral analysis and sleep staging, the integration of causal inference approaches—including time-lagged MIC or transfer entropy—in future investigations would enable more rigorous determination of directional associations between variables.

## 5 Conclusion

In summary, the present study successfully implemented a non-invasive, dynamic assessment of brain-heart-gut coupling during sleep by synchronously acquiring EEG, ECG and BS signals. The results demonstrated that the strength of brain-heart-gut coupling varied across different sleep stages, exhibiting a gradual attenuation as sleep deepened. A notable finding was that HGC exhibited greater strength compared to BGC. In addition, EEG-beta coupling exhibited distinct sleep-stage dependency, while EEG-delta band demonstrated enhanced coupling strength specifically during NREM sleep. Collectively, these findings imply that the use of sleep EEG, ECG and BS signals provides an effective approach for evaluating brain-heart-gut coupling during sleep. Furthermore, this study extends the dynamic assessment of brain-heart-gut coupling to sleep research, which not only facilitates the elucidation of interaction patterns between the central nervous system and peripheral organ systems across different sleep stages, but also provides a significant methodological framework for investigating the mechanisms underlying sleep-related disorders and functional gastrointestinal diseases.

## Data Availability

The raw data supporting the conclusions of this article will be made available by the authors, without undue reservation.
